# Does adjuvant hepatic artery infusion chemotherapy improve patient outcomes for hepatocellular carcinoma following liver resection? A meta-analysis

**DOI:** 10.1186/s12957-023-03000-1

**Published:** 2023-04-03

**Authors:** Lingbo Hu, Yu Zheng, Jiangyin Lin, Xingpeng Shi, Aidong Wang

**Affiliations:** 1 Department of Hepatopancreatobiliary Surgery, Taizhou Hospital of Zhejiang Province Affiliated to Wenzhou Medical University, Zhejiang, China; 2 Department of Hepatopancreatobiliary Surgery, Taizhou Enze Medical Center (Group), Enze Hospital, Zhejiang, China; 3 Department of Rehabilitation Medicine, the First Affiliated Hospital of Nanjing Medical University, Jiangsu, China; 4 Department of Blood Purification, Taizhou Hospital of Zhejiang Province Affiliated to Wenzhou Medical University, Zhejiang, China

**Keywords:** Hepatic artery infusion, Hepatectomy, Hepatocellular carcinoma, Portal vein thrombus, Chemotherapy, Meta-analysis

## Abstract

**Background:**

Adjuvant hepatic artery infusion chemotherapy (HAIC) has been shown to be beneficial to the patient outcomes in hepatocellular carcinoma (HCC).

**Methods:**

Randomized controlled trials (RCTs) and non-RCTs were identified from six databases up to January 26, 2023. Patient outcomes were assessed using overall survival (OS) and disease-free survival (DFS). Data were presented as hazard ratios (HR, 95% confidence intervals, or CIs).

**Results:**

The present systematic review included 2 RCTs and 9 non-RCTs with a total of 1290 cases. Adjuvant HAIC improved OS (HR of 0.69; 95% CI of 0.56–0.84; *p* < 0.01) and DFS (HR of 0.64; 95% CI of 0.49–0.83; *p* < 0.01). Subgroup analysis showed that HCC patients with portal vein invasion (PVI) or microvascular invasion (MVI) benefit from adjuvant HAIC in terms of OS ((HR of 0.43; 95% CI of 0.19–0.95; *p* < 0.01) and (HR of 0.43; 95% CI of 0.19–0.95; *p* = 0.0373), respectively) and DFS ((HR of 0.38; 95% CI of 0.21–0.69; *p* < 0.01) and (HR of 0.73; 95% CI of 0.60–0.88; *p* = 0.0125), respectively). Adjuvant HAIC with the oxaliplatin-based approach significantly improved OS (HR of 0.60; 95% CI of 0.36–0.84; *p* = 0.02) and (HR of 0.59; 95% CI of 0.43–0.75;* p* < 0.01), respectively).

**Conclusion:**

This meta-analysis demonstrated that postoperative adjuvant HAIC was beneficial in HCC patients with PVI and MVI. It remains unclear whether HAIC can improve the survival outcome in all HCC patients after hepatic resection.

**Supplementary Information:**

The online version contains supplementary material available at 10.1186/s12957-023-03000-1

## Introduction

Liver cancer is the 4th most common cause of cancer-related death worldwide with hepatocellular cancer (HCC) accounting for most primary liver cancer cases [1]. Liver resection (LR) is a common treatment approach for HCC [2] that exhibits a high recurrence rate of up to 70% and poor survival in the long term [3]. Fortunately, adjuvant strategies had been shown to decrease the recurrence and enhance the survival rate [4].

Current adjuvant strategies include transarterial chemoembolization (TACE), hepatic artery infusion chemotherapy (HAIC), antiviral therapy, and radiation therapy [5]. The ability of TACE in reducing HCC recurrence and improving the overall survival (OS) and disease-free survival (DFS) had been backed by several randomized controlled trials (RCTs) and meta-analyses [6–9]. Mechanistically similar to TACE, HAIC is another adjuvant strategy that also holds several advantages over TACE. By delivering a higher concentration of chemotherapeutics (compared to TACE) via the hepatic artery to the liver with continuous infusion, HAIC extends the exposure of residual cancer cells to chemotherapeutic reagents. In addition, HAIC does not embolize the hepatic artery, avoiding damages to the residual liver that are commonly seen in TACE. The efficacy and safety of HAIC had been investigated and it had been documented that HAIC results in better outcomes for patients with advanced HCC [10, 11]. However, its efficacy as adjuvant therapy for patients who received liver resection for HCC is still unclear.

Although the use of HAIC in reducing tumor recurrence after liver resection was reported as early as 1990s [12], inconsistent results had been documented in studies with different patient and tumor characteristics as well as different chemotherapy regimens [13–17]. Many early studies on the efficacy of HAIC for patients with HCC after liver resection (before 2015) were limited by the sample size. Several studies were published after 2017 to further explore this topic [18–22]. For comprehensive analysis, Moran et al. reported that adjuvant hepatic arterial infusion with chemotherapy or 131 Iodine lipiodol improves both the OS and DFS after LR, especially in patients with HCC ≥ 7 cm [23]. Li et al. and Ke et al. also concluded that adjuvant HAIC improves both the OS and DFS after LR [24, 25], and Liu et al. pointed out that HAIC is the most effective adjuvant regimen after surgical resection for HCC [4]. However, these meta-analyses limited by the small number of literatures or included literatures with a sample size. To clarify whether HAIC would benefit patients with radical liver resection, we performed a meta-analysis with extensive searching and excluding studies with small sample sizes in this study. The impact of HAIC on improving patient survival after liver resection was systematically assessed.

## Methods

The proposed review is registered in PROSPERO (registration no. CRD42023400918).

### Search strategy

Six databases (PubMed, Embase, Web of Science, Scopus, Ovid, and Cochrane library) were searched for English articles till January 26, 2023. For PubMed, the following keywords and MeSh terms were used: hepatectomy, liver resection, hepatic artery infusion chemotherapy or HAIC, hepatocellular carcinoma or HCC. Details of search strategy of all databases were shown in Supplementary materials 1. References in the identified studies were further searched manually for more studies.

### Inclusion criteria

The following criteria were applied: RCTs and non-RCTs in English; liver surgery with or without HAIC in treating HCC patients; OS and/or DFS were reported.

### Exclusion criteria

Non-comparative studies, conference abstracts, case reports, and reviews were excluded. For studies with overlapped patient cohorts, only the top one (highest quality, largest sample size, or most recent) was included while all the others were excluded. Studies with a sample size of < 10 in each group were excluded.

### Quality assessment and data extraction

Initial assessment of the quality of each study and the following data extraction were conducted independently by two researchers (LB Hu and XP Shi). For nonrandomized comparative trials, the Newcastle–Ottawa scale (NOS) with a scale up to 9 points was used (5 or less for low quality; 6–7 for medium quality; and 8 or more for high quality) for quality assessment. The risk of bias for RCTs was assessed according to the Cochrane Collaboration [26].

Predesigned and standardized forms were used to extract study details (e.g., first author, country, year of publication, patient information, tumor characteristics, and chemotherapy treatment). The primary outcomes including OS, DFS, survival rates (1, 3, and 5 years) as well as the DFS rate were extracted either directly from the original reports or indirectly from estimation with the Kaplan–Meier curve using the Engauge Digitizer software (version 4.1). Any disagreements between the two independent analyses were resolved by a third researcher (AD Wang).

### Statistical analyses

The inverse variance method was used for determining the hazard ratio (HR) and 95% confidence interval (CI) values. The Mantel–Haenszel method was employed for determining the risk ratio (RR) and 95% CI values. Heterogeneity was assessed using the *χ*^2^ method (*I*^2^ of 25% for low heterogeneity; 50% for moderate heterogeneity). The selection of the test model was based on the heterogeneity level with the random-effects model for *I*^2^ > 50% [27]. The robustness of the conclusion was assessed by sensitivity analysis. Subgroup analysis was based on the status of portal vein invasion (PVI), microscopic vein invasion (MVI), and the chemotherapy regimen. The publication bias was determined using funnel plots with Begg’s test and Egger’s test. Statistical significance was determined using a *p* value cutoff of 0.05. Pooled results were assessed using the Grading of Recommendations, Assessment, Development, and Evaluation (GRADE) approach that takes into consideration of inconsistency, imprecision, indirectness, and the risk of bias. Most of statistical computations were conducted in R (v4.0.3), a fraction of them were conducted in Stata (v15.1).

## Results

### Study search and selection

**Fig. 1:**
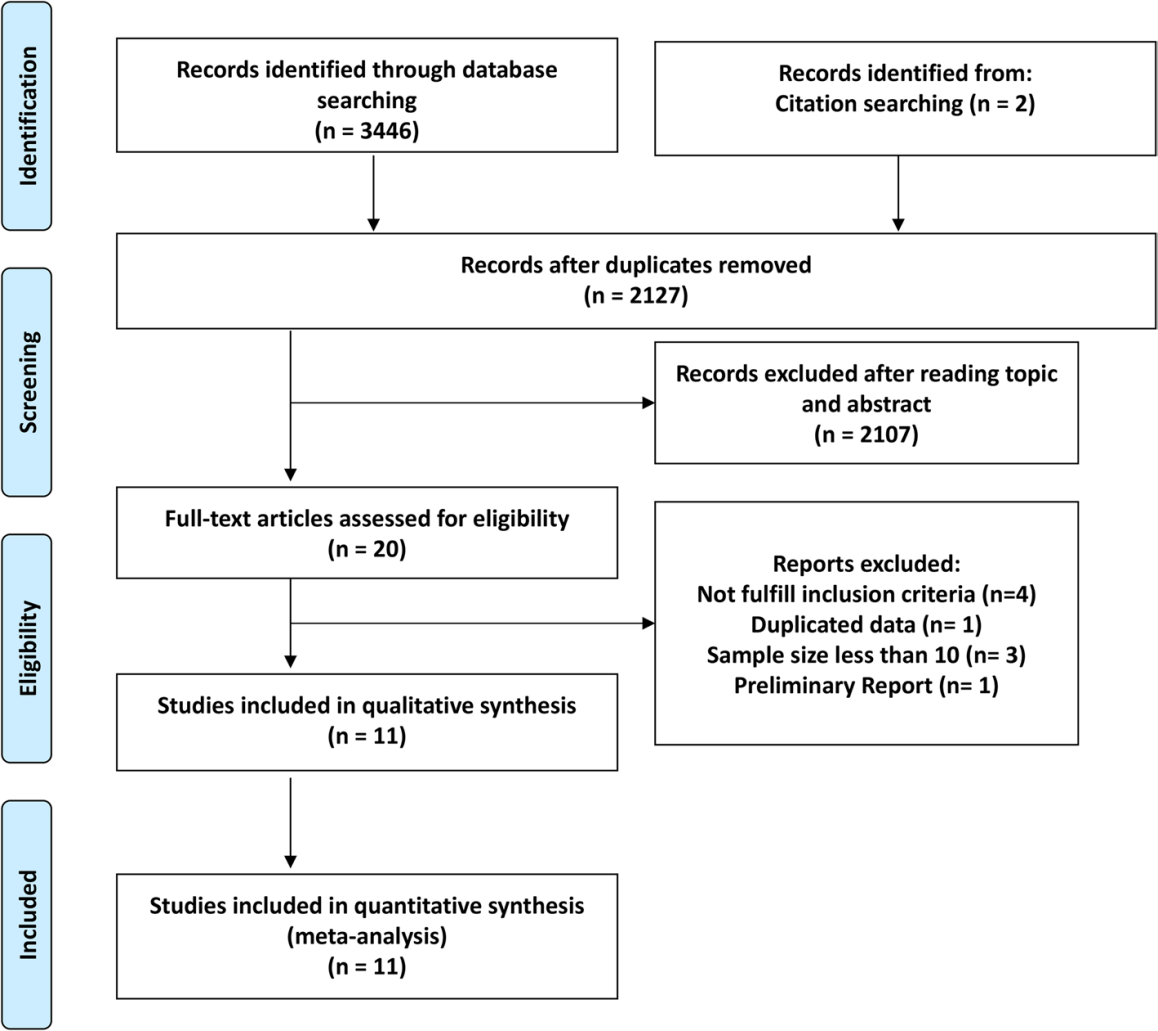
Flow chart of study selection

Database searching yielded a total of 2127 entries with 2107 excluded after reviewing the title and abstract (Fig. 1). For the remaining articles, eight were further excluded because they did not meet the inclusion criteria (*n* = 4), had a small sample size (*n* = 3), used the same dataset (*n* = 1), or reported preliminary result (*n* = 1). Thus, 11 studies were selected for the meta-analysis [13–21, 28, 29].

### Study characteristics

The included 11 studies consisted of two RCTs and nine retrospective studies, involving a total of 1290 patients with 513 patients treated with LR and adjuvant HAIC (the HAIC group) and 777 patients treated with only LR (the LR-only group). These studies were from Japan (*n* = 7), China (*n* = 3), and Korea (*n* = 1).

Patient characteristics are shown in Table 1. Patients in these studies showed a wide range of PVI from 0% (*n* = 4), 25.8% to 84.8% (*n* = 3), to 100% (*n* = 3). Five studies reported the status of MVI. Chemotherapy regimens included 5-fluorouracil (5-FU) in combination with interferon (*n* = 2 from Japan), cisplatin alone or in combination with 5-FU and other drugs (*n* = 4 from Japan and *n* = 1 from Korea), 5-FU and oxaliplatin (*n* = 2 from China), and 5-FU, cisplatin, leucovorin, and epirubicin (*n* = 1 from China). The OS ranged from 33.2 to 56.4 months in the HAIC group and 8.5 to 56.9 months in the LR-only group. The DFS ranged from 10.5 to 50.6 months in the HAIC group and 5.5 to 54.5 months in the LR-only group.

**Table 1:** Characteristics of included studies

Study	Design	NOS	Group	Sample size	Tumor size, cm	Number of tumor S/M	Tumor differentiation well/moderately/poorly	Surgical margin N/P	Portal vein invasion yes/no	MVI yes/no	median OS (month)	median DFS (month)	Chemotherapy
Nomami	R	6	LR + HAIC	19	NA	NA	NA	NA	NA	NA	NA	NA	5-FU + DO + MI
1991 Japan			LR alone	113	NA	NA	NA	NA	NA	NA	NA	NA	
Kim	R	7	LR + HAIC	31	4.8 ± 2.3	29/2	0/10/14	8/23	11/20	25/6	NR	10.5	5-FU + CI
2011 Korea			LR alone	62	4.3 ± 2.4	60/2	1/20/34	23/39	13/49	43/19	NR	7.5	
Kumatoto	R	9	LR + HAIC	16	7.61 ± 4.34	NA	NA	NA	16/0	NA	55.3	12.2	5‐FU + Interferon
2013 Japan			LR alone	17	9.48 ± 6.54	NA	NA	NA	12/5	NA	16.1	6.9	
Nagano	R	7	LR + HAIC	30	NA	NA	NA	NA	30/0	NA	36.5	29	5‐FU + Interferon
2013 Japan			LR alone	20	NA	NA	NA	NA	20/0	NA	8.5	5.5	
Nitta	R	8	LR + HAIC	38	6.66 ± 3.93	13/25	14/23/1	33/5	38/0	NA	55	13	5-FU + CI/5-FU + CI + MI
2013 Japan			LR alone	35	7.00 ± 4.23	12/23	22/12/1	32/3	35/0	NA	26	7	
Kojima	R	9	LR + HAIC	27	7.0 (2.8–18.0)	10/17	1/12/12	19/8	27/0	NA	33.2	12.4	5-FU + CI + EP
2015 Japan			LR alone	25	5.0 (1.4–17.0)	8/17	3/9/12	19/6	25/0	NA	21.5	6.2	
Feng	R	9	LR + HAIC	42	6.2 ± 1.5	24/18	10/12/20	NA	0/42	28/14	51.1	42.2	5-FU + OX + MI
2017 China			LR alone	43	5.7 ± 1.3	23/20	12/14/17	NA	0/43	25/18	32.8	22.1	
Hsiao	R	7	LR + HAIC	61	20(<= 5 cm) 41(> 5 cm)	33/28	0/16/45	33(< 10 mm) 28 (≥ 10 mm)	0/61	NA	56.4	50.6	5-FU + CI + LE + EP
2017 China			LR alone	160	81(<= 5 cm) 79(> 5 cm)	81/79	0/56/104	121(< 10 mm) 39 (≥ 10 mm)	0/160	NA	56.9	54.5	
Hamada	R	7	LR + HAIC	37	5.4 ± 3.6	NA	7/30 (well/other)	NA	37/0	37/0	NR	22.7	CI
2020 Japan			LR alone	85	5.7 ± 3.7	NA	17/68 (well/other)	NA	52/33	62/23	NR	10.5	
Hirokawa	RCT	/	LR + HAIC	55	3.4 (1.0–14.5)	41/14	46/9 (other/poorly)	5 (0–32) mm	0/55	14/41	NR	44.9	CI + LI
2020 Japan			LR alone	59	3.1 (1.0–12)	51/8	14/45 (other/poorly)	5 (0–36) mm	0/59	11/48	NR	35	
Li	RCT	/	LR + HAIC	157	5.6 (1.8–30.0)	114/45	4/59/92	NA	0/157	157/0	NR	20.3	FOLFOX
2022 China			LR alone	158	5.4 (1.5–16.0)	128/30	2/76/80	NA	0/158	158/0	NR	10.0	

*R* retrospective study, *RCT* randomized controlled trial, *NOS* Newcastle–Ottawa Scale, *LR* hepatic resection, *HAIC* hepatic artery infusion chemotherapy, *NA* not available, *NR* not reached, *S* solitary, *M* multiple, *N* negative, *P* positive, *MVI* microvascular invasion, *OS* overall survival, *DFS* disease-free survival, *5-FU* 5-fluoruracil, *DO* doxorubicin, *CI* cisplatin, *OX* oxaliplatin, *MI* mitomycin-C, *LE* Leucovorin, *EP* Epirubicin, *LI* Lipiodol

### Quality assessment

The risk of bias of RCTs was low. Details of quality assessment of these studies were shown in Supplementary materials 2. For the nine retrospective studies, four were of high quality and five were of medium quality (Table 1).

### Overall survival

**Fig. 2:**
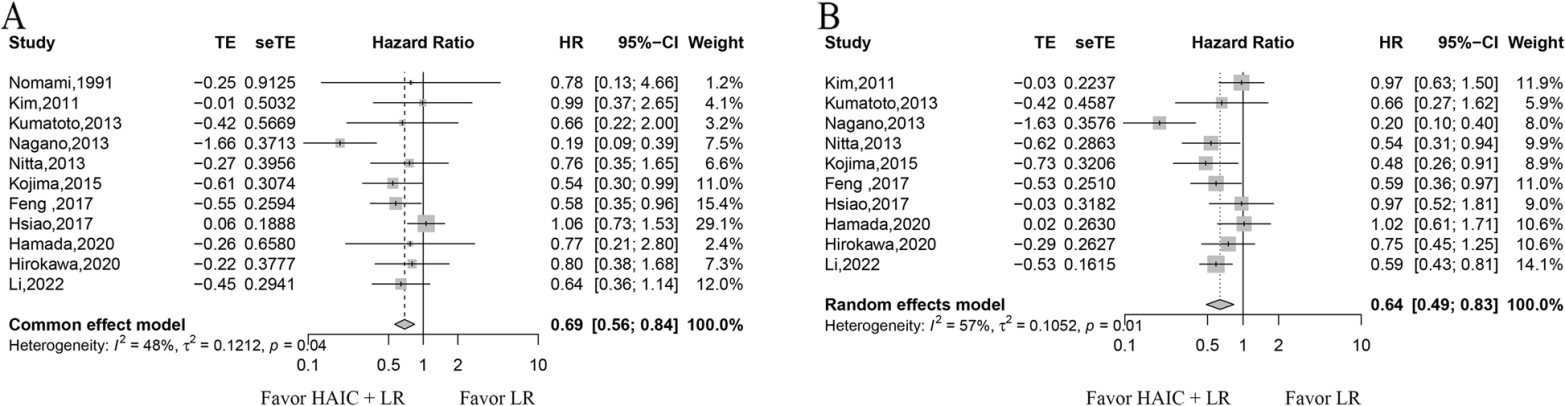
The overall survival and disease-free survival. Forest plots showing the overall survival and disease-free survival are shown in **A** and **B**, respectively

**Fig. 3:**
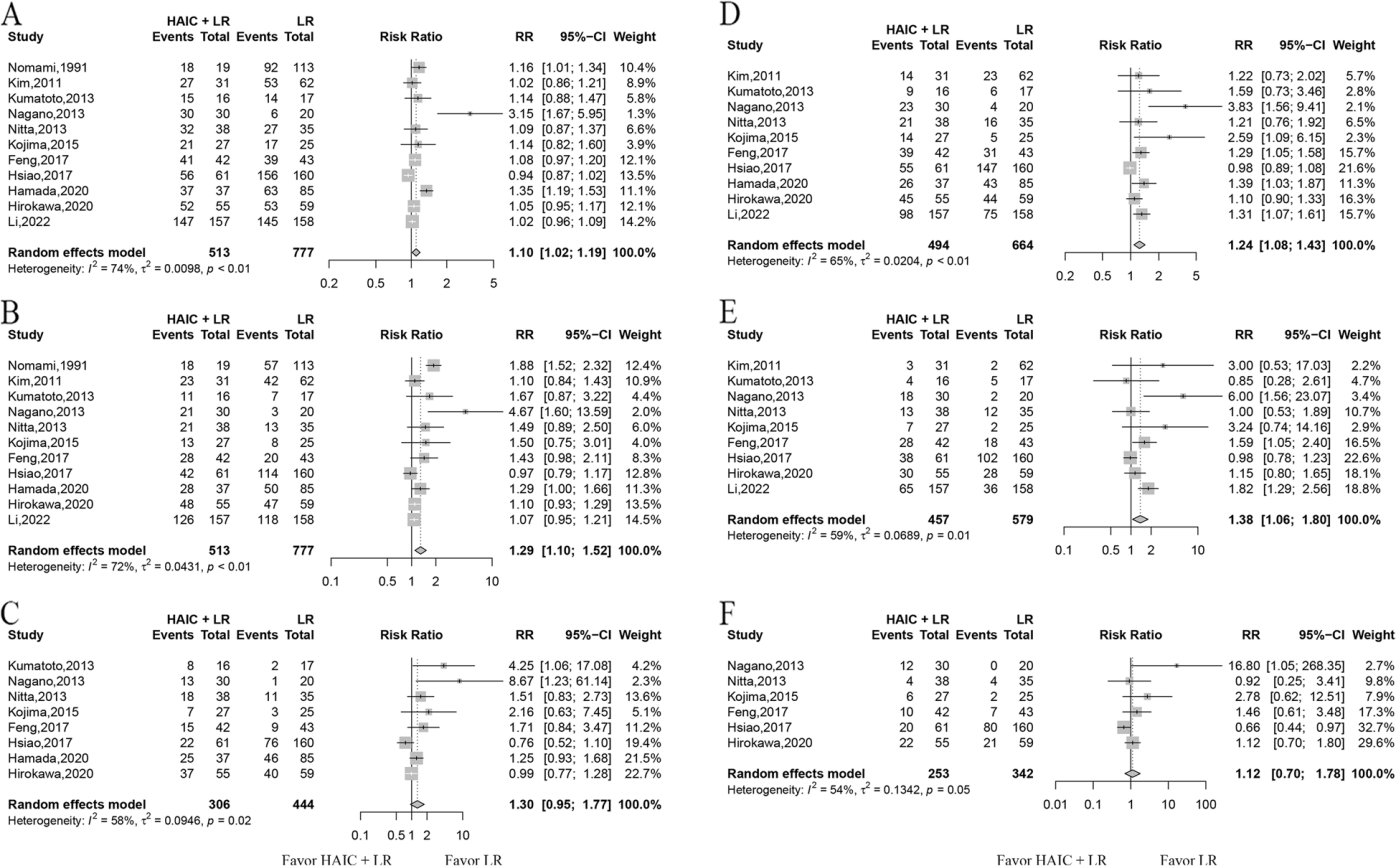
The overall survival and disease-free survival rates. Forest plots showing the 1-, 3-, and 5-year overall survival rates of the HAIC and LR-only groups are shown in **A** to **C**, respectively. Forest plots showing the 1-, 3-, and 5-year disease-free survival rates of the HAIC and LR-only groups are shown in **D** to **F**, respectively

Median survival time, available in six studies, ranged from 36.5 to 56.4 months in the HAIC group and 8.5 to 56.9 months in the LR-only group (Table 1). HR of OS were available for all studies, the fixed effect model was used, pooled data showed that HAIC improved OS (HR of 0.69; 95% CI of 0.56–0.84; *p* < 0.01) (Fig. 2). While the survival rate at 1 and 3 years were available for all studies, that 5-year was only obtained in eight studies. Since significant heterogeneity among studies at 1-year, 3-year, and 5-year survival was found, the random effect model was used to pool these studies. HAIC improved the 1-year (RR of 1.10; 95% CI of 1.02–1.19; *p* < 0.01) and 3‐year survival (RR of 1.29; 95% CI of 1.10–1.52; *p* < 0.01). No difference at 5-year survival was found between the two groups (RR of 1.30; 95% CI of 0.95–1.77; *p* = 0.1032) (Fig. 3).

### Disease-free survival rates

Median DFS time, available in ten studies, ranged from 10.5 to 50.6 months in the HAIC group and 5.5 to 54.5 months in the LR-only group. Since significant heterogeneity among studies on DFS was found, the random effect model was used to pool these studies. HR of DFS were available for ten studies, pooled data showed that HAIC improved DFS (HR of 0.64; 95% CI of 0.49–0.83; *p* < 0.01) (Fig. 2). The DFS data at 1-year, 3-year, and 5-year were only obtained in ten, nine, and six studies, respectively. HAIC improved the 1-year (RR of 1.24; 95% CI of 1.08–1.43; *p* < 0.01) and 3-year (RR of 1.38; 95% CI of 1.06–1.80; *p* = 0.0186), respectively. No difference at 5-year (RR of 1.12; 95% CI of 0.70–1.78; *p* = 0.6454) DFS was found between the two groups (Fig. 3).

### HAIC-related adverse effect

The details of HAIC-related adverse effects are shown in Table 2. Fever, nausea or vomiting, diarrhea, neutropenia, and hepatic toxicity are common, mild, and easily controlled. Nitta et al. reported that three patients had grade 3 vomiting, and three had severe neutropenia, Kojima et al. reported one grade 3 myelosuppression, and Hirokawa et al. reported that three patients had grade 3 hepatic toxicity. Li et al. reported 104 grade 1–2 adverse effects and 2 grade 3–4 adverse effects.

**Table 2:** Complication related to HAIC

Study	Complication
Nomami 1991	No serious complications attributable to the adjuvant arterial chemotherapy were observed. Some patients complained of transient fever or uncomfortable feelings after the injection of doxorubicin, mitomycin C, and lipiodol
Kim 2011	No serious systemic adverse events (AEs) was observed
Kumatoto 2013	A total of 6.3% of patients experienced grade 3 decreases in white blood cell and neutrophil counts
Nagano 2013	Easily controlled fever was common, but no serious systemic AEs was observed
Nitta 2013	A total of 7.9% of patients experienced grade 3/4 neutropenia and vomiting
Kojima 2015	One developed grade 2 acute kidney injury and one had persistent grade 3 myelosuppression
Feng 2017	Paresthesia (14 of 42), neutropenia (15 of 42), thrombocytopenia (16 of 42), anemia (10 of 42), nausea or vomiting (30 of 42), diarrhea (8 of 42), and hepatic toxicity (25 of 42). All toxicities and complications were controlled
Hsiao 2017	Nausea, vomiting, and mild AST/ALT elevation were noted, but no serious AEs was observed
Hamada 2020	Not mentioned
Hirokawa 2020	A total of 5.4% of patients experienced grade 3 AST/ALT elevation. No grade 4 AEs was observed
Li 2022	A total of 82, 22, and 2 patients experienced grade 1, grade 2, and grade 3–4 AEs, respectively

*HAIC* hepatic arterial infusion chemotherapy, *ALT* alanine transaminase *AST* aspartate aminotransferase

### Subgroup analysis

Subgroup analysis based on the PVI status was conducted. In HCC patients with PVI, adjuvant HAIC significantly improved OS (HR of 0.43; 95% CI of 0.19–0.95; *p* < 0.01), 3-year (RR of 1.86; 95% CI of 1.09‐3.19; *p* < 0.01) and 5-year survival rate (RR of 1.97; 95% CI of 1.02–3.79; *p* < 0.01). What is more, adjuvant HAIC showed a significant impact at DFS (HR of 0.38; 95% CI of 0.21–0.69; *p* < 0.01) and 1-year (RR of 2.10; 95% CI of 1.03–4.29; *p* < 0.01) (Tables 3 and 4). For HCC patients without portal vein invasion, adjuvant HAIC significantly improved DFS (HR of 0.66; 95% CI of 0.53‐0.82; *p* < 0.01), but it did not show improvement on the OS and DFS at 1, 3, or 5 years.

**Table 3:** Subgroup analysis of OS

Subgroup	No. of study	Outcomes	RR	95%CI	*I*^2^
PVI					
Without PVI	4	OS^a^	0.81	0.63–1.03	32%
	4	1y-OS	1.01	0.96–1.07	60%
	4	3y-OS	1.08	0.99–1.18	10%
	3	5y-OS	0.98	0.72–1.34	52%
With PVI	3	OS^a^	0.43	0.19–0.95	73%
	3	1y-OS	1.49	0.81–2.75	79%
	3	3y-OS	1.94	1.31–2.87	47%
	3	5y-OS	1.97	1.02–3.79	31%
Chemotherapy					
Cisplatin based	7	OS^a^	0.78	0.56–1.00	0%
	7	1y-OS	1.10	0.99–1.21	76%
	7	3y-OS	1.26	1.04–1.54	76%
	5	5y-OS	1.08	0.85–1.38	44%
Interferon + 5-FU	2	OS^a^	0.20	0.05–0.35	1%
	2	1y-OS	1.81	0.67–4.91	88%
	2	3y-OS	2.55	0.95–6.88	61%
	2	5y-OS	5.40	1.74–16.77	0%
Oxaliplatin based	2	OS^a^	0.60	0.36–0.84	0%
	2	1y-OS	1.03	0.98–1.09	0%
	2	3y-OS	1.17	0.90–1.51	49%
	1	5y-OS	1.71	0.84–3.47	/
MVI					
With MVI	5	OS^a^	0.68	0.50–0.92	0%
	5	1y-OS	1.09	0.99–1.21	75%
	5	3y-OS	1.12	1.03–1.22	0%
	3	5y-OS	1.17	0.97–1.42	27%

^a^ Data present as hazard ratio and 95% confidence interval

*PVI* portal vein invasion, *MVI* microvascular invasion, *5-FU* 5-fluorouracil, *1-y OS* 1-year overall survival, *3-y OS* 3 years overall survival, *5-y OS* 5 years overall survival, *OR* odds ratio, *CI* confidence interval

**Table 4:** Subgroup analysis of DFS

Subgroup	No. of study	Outcomes	RR	95%CI	*I*^2^
PVI					
Without PVI	4	DFS^a^	0.66	0.53–0.82	0%
	4	1y-DFS	1.14	0.98–1.32	71%
	4	3y-DFS	1.31	0.981.77	72%
	3	5y-DFS	0.94	0.59–1.49	56%
With PVI	3	DFS^a^	0.38	0.21–0.69	63%
	3	1y-DFS	2.10	1.03–4.29	68%
	3	3y-DFS	2.33	0.75–7.30	70%
	3	5y-DFS	2.37	0.62–9.08	47%
Chemotherapy					
Cisplatin based	6	DFS^a^	0.71	0.52–0.90	20%
	6	1y-DFS	1.15	0.98–1.35	52%
	5	3y-DFS	1.05	0.88–1.26	7%
	4	5y-DFS	0.94	0.59–1.50	45%
Interferon + 5-FU	2	DFS^a^	0.30	0–0.69	41%
	2	1y-DFS	2.40	1.02–5.65	52%
	2	3y-DFS	2.18	0.32–14.76	79%
	1	5y-DFS	16.80	1.05–268.35	/
Oxaliplatin based	2	DFS^a^	0.59	0.43–0.75	0%
	2	1y-DFS	1.24	1.08–1.43	0%
	2	3y-DFS	1.72	1.32–2.24	0%
	1	5y-DFS	1.46	0.61–3.48	/
MVI					
With MVI	5	DFS^a^	0.73	0.60–0.88	30%
	5	1y-DFS	1.24	1.12–1.38	0%
	4	3y-DFS	1.57	1.27–1.94	25%
	2	5y-DFS	1.19	0.79–1.81	0%

^a^ Data present as hazard ratio and 95% confidence interval

*PVI* portal vein invasion, *MVI* microvascular invasion, *5-FU* 5-fluorouracil, *1-y DFS* 1-year disease-free survival, *3-y DFS*, 3 years disease-free survival, *5-y DFS* 5 years disease-free survival, *OR* odds ratio, *CI* confidence interval

Subgroup analysis based on the MVI status was conducted. In HCC patients with MVI,adjuvant HAIC significantly improved OS (HR of 0.43; 95% CI of 0.19–0.95; *p* = 0.0373) and 3-year survival (RR of 1.12; 95% CI of 1.03–1.22; *p* < 0.01). What is more, adjuvant HAIC showed a significant impact at DFS (HR of 0.73; 95% CI of 0.60‐0.88; *p* = 0.0125), 1-year (RR of 1.24; 95% CI of 1.12–1.38; *p* < 0.01), and 3-year (RR of 1.57; 95% CI of 1.27–1.94; *p* < 0.01) (Tables 3 and 4).

Subgroup analysis was also performed based on three chemotherapeutic regimens: cisplatin-based, oxaliplatin-based, and 5-FU and interferon-based. While adjuvant HAIC with the oxaliplatin-based approach significantly improved OS (HR of 0.60; 95% CI of 0.36–0.84; p = 0.02), the 5-FU/interferon regimen significantly improved OS (HR of 0.60; 95% CI of 0.36–0.84; *p* < 0.01) and the 5-year survival rate (RR of 5.40; 95% CI, 1.74–16.77; *p* < 0.01), the cisplatin-based approach significantly improved the 3-year survival rate (RR of 1.26; 95% CI, 1.04–1.54; *p* < 0.01) (Table 3). Remarkably, all approaches significantly improved DFS (HR of 0.59; 95% CI of 0.43–0.75; *p* < 0.01 for the oxaliplatin-based approach; HR of 0.71; 95% CI of 0.52–0.90; *p* < 0.01 for the cisplatin-based approach; and HR of 0.30; 95% CI of 0–0.69; *p* < 0.01 for the 5-FU/interferon approach).

### Publication bias

Publication bias was not detected based on Begg’s test and Egger’s test (Supplementary materials 3).

### Sensitivity analysis

Sensitivity analysis was performed by excluding each study in turn and combining HR for the remaining included studies. Sensitivity analysis showed that a certain heterogeneity between the results of “Nagano2013” and other studies. However, both OS and DFS were robust (Supplementary materials 4). As a study conducted by Nomami et al. was published too early in 1991, potential heterogeneity may exist. Sensitivity analysis showed that a certain heterogeneity between the results of “Nagano2013” and other studies. Hence, we re-calculated by omitting study “Nomami 1991” and “Nagano2013” successively and omitting both of them. The results still showed that HAIC improved bothe OS and DFS (Supplementary materials 5).

### GRADE evidence

The GRADE evidence profile was summarized in Supplementary materials 6. GRADE analysis showed that the quality of evidence for OS and DFS outcomes with or without HAIC after LR for HCC was very low.

## Discussion

Our meta-analysis provides the first analysis of the efficacy of HAIC on the outcomes of HCC patients after LR. The results indicated that adjuvant HAIC after LR improved OS and DFS. Subgroup analysis revealed that HCC patients with PVI could benefit from HAIC. Both the oxaliplatin-based and 5-FU/interferon regimens were more effective than the cisplatin-based chemotherapy on OS. Thus, an appropriate chemotherapy regimen for a specific patient subgroup should be recognized to achieve better outcomes.

Ke et al. conducted a meta-analysis on this topic recently, and the conclusion was similar with ours [24]. However, they included three studies with sample size less than 10 in each group wich may affect the reliability of results. Considering this issue, we excluded these studies and included a most recent large sample studied to make the results more reliable. What is more, publication bias existed and the “trim and fill” analysis suggested that the unpublished studies might have few effects on the results. Li et al. concluded that HAIC improves both the OS and DFS for patients with advanced HCC as primary treatment or patients with resectable HCC as adjuvant treatment [25]. However, only five studies assessed the role of adjuvant HAIC for patients with resectable HCC, studies published in 2020 and 2021 were not included. Subgroup analysis could not be performed due to the small number of literatures included.

Moran et al. reported that adjuvant hepatic artery infusion therapy including HAIC and hepatic artery infusion with I‐131 lipiodol could improve both OS and DFS for HCC patients [23]. Similarly, our study found HAIC has a positive effect on the short and mid-term OS rates and the short-term DFS rate. However, we found that the 5-year survival and DFS were comparable between the HAIC and the LR-only groups. The discrepancy could be attributed to the small sample size in the previous study. This is further backed by our sensitivity analysis for the 5-year survival rate, which showed that the significance of HAIC is only observed after removing the study with the largest sample size. Thus, future studies should enroll more samples to consolidate this finding.

It had been reported that TACE could significantly reduce tumor recurrence and improve DFS (hazard ratio of 0.68; 95% CI, 0.49–0.93) and OS (hazard ratio of 0.59; 95% CI, 0.36–0.97) for patients with hepatitis virus B related HCC who have a high risk of recurrence after LR [7]. In addition, TACE significantly increased the median OS from 22.37 to 44.29 months and the median DFS from 9.27 to 17.45 months in patients with single HCC and microvascular invasion (MVI) [6]. Additionally, adjuvant TACE improved the survival of HCC patients with portal vein tumor thrombus after hepatectomy [30]. A previous meta-analysis showed that adjuvant TACE was beneficial in HCC patients with multinodular tumors, tumor diameter > 5 cm, or MVI-positive. Similarly, Niizeki et al. demonstrated the benefit of adjuvant HAIC treatment for HCC patients with MVI [31]. Such a benefit of adjuvant HAIC had also been reported for HCC patients with tumor thrombosis [32]. Our analysis demonstrated that patients with HCC could benefit from HAIC in the short term and that HCC patients with PVI or MVI benefit from HAIC in the long term. A high probability of tumor transfer via the portal vein before liver resection could lead to a high likelihood of persistent tumor cells after LR in HCC patients with PVI, HCC complicated with MVI is prone to have residual cancer cells after hepatectomy, making the chemotherapy more evident. It was reported that gamma knife radiosurgery and transcatheter arterial chemoembolization had different survival effect for patients with HCC depending on the type of portal vein tumor thrombus (PVTT) [33]. Hence, it would be better to evaluate the efficacy of HAIC for HCC patients based on the type of PVTT in further research.

Various chemotherapeutic regimens (antitumor antibiotics, antibiotics + platinum agents, antibiotics + anti-metabolic agents, antibiotics + platinum, antimetabolic agents, and platinum + anti-metabolic agents) had been used for TACE and HAIC [9]. However, which regimen is more effective remains unclear. While one RCT showed that HAIC and low dose of cisplatin and 5-FU did not improve the OS in patients with advanced HCC [34], HAIC with oxaliplatin + 5-FU/leucovorin may have a potential benefit of survival for such patients [35]. In addition, radiotherapy and/or sorafenib with HAIC of FOLFOX regimen improved the OS in HCC patients with PVI [36, 37]. Similarly, we observed that HAIC of oxaliplatin-based agents improved the OS while cisplatin-based agents did not. Thus, oxaliplatin may be more suitable for HCC. For the 5-FU + interferon approach, it is unclear which drug could improve the survival rate. On the other hand, Li et al. showed that HAIC of mFOLFOX regimen had higher objective response rate and improved the OS and DFS than conventional TACE did [10]. Besides, a RCT by Li et al. demonstrated that HAIC of FOLFOX regimen is superior to TACE on OS, DFS and objective response rate [38]. Hence, despite the lack of direct evidence, we suppose that FOLFOX or mFOLFOX regimen is a suitable regimen for HAIC.

In regard to complication of HAIC, only nine grade 3/4 complication was reported, besides, tolerable nausea, vomiting, or myelosuppression are common. What is more, both study mentioned before showed that serious adverse events were higher in the TACE group than in the HAIC group. Hence, HAIC is a safe therapy.

There are several limitations in this study. First, strong publication bias and significant heterogeneity limited the power of meta-analysis. The heterogeneity issue was partially resolved by subgroup analysis based on either the PVI status or the chemotherapy. The results showed that the heterogeneity was attributed to the patient cohort, the geographical area, inclusion criteria of different studies, and the chemotherapy regimen. Interestingly, we found that HAIC was more effective in HCC patients with PVI or MVI who with high risk of recurrence. Second, publication bias was significant as positive results are more likely to be published, which in turn affects the result of the meta-analysis. Third, only two RCTs were included here with low-GRADE evidence profiles for both OS and DFS. Fourth, the sample size in each study was relatively small. Fifth, all studies included were from Asia so that the conclusion may not be applicable in western countries (Supplementary materials 7).

## Conclusion

Our meta-analysis showed that HAIC improves OS and DFS. Notably, HCC patients with PVI or MVI may benefit from adjuvant HAIC. However, the benefit of HAIC in HCC patients in general is still not clear due to significant heterogeneity, and very low quality of GRADE evidence profile. Thus, large and well-designed RCTs considering risk of recurrence and chemotherapeutic regimens are needed for future studies. In addition, a standard and effective chemotherapeutic regimen is needed.

## Supplementary Information

**Additional file 1: Supplementary materials 1.** Search strategy. **Supplementary materials 2.** The differences of included studies between our and previous studies. **Supplementary materials 3.** Risk assessment of RCTs. **Supplementary materials 4.** Plot of publication bias. **Supplementary materials 5.** Plot of sensitivity analysis. **Supplementary materials 6.** Plot of OS and DFS after omitting some studies. **Supplementary materials 7.** GRADE analysis of OS and DFS in the HAIC group.

## Data Availability

The datasets used and/or analyzed during the current study are available from the corresponding author on reasonable request.

## Abbreviations

HCC: Hepatocellular cancer
LR: Liver resection
TACE: Transarterial chemoembolization
HAIC: Hepatic artery infusion chemotherapy
OS: Overall survival
DFS: Disease-free survival
RCTs: Randomized controlled trials
NOS: Newcastle-Ottawa scale
RR: Risk ratio
CI: Confidence interval
PVI: Portal vein invasion
MVI: Microscopic vein invasion
5-FU: 5-Fluorouracil
PVTT: Portal vein tumor thrombus
