# Supplementary material for: Does adjuvant hepatic artery infusion chemotherapy improve patient outcomes for hepatocellular carcinoma following liver resection? A meta-analysis
Source: World J Surg Oncol. 2023 Apr 3;21:121. doi: 10.1186/s12957-023-03000-1 (PMC10069128; doi:10.1186/s12957-023-03000-1)
Supplement: Supplementary file 1 — Additional file 1: Supplementary materials 1. Search strategy. Supplementary materials 2. The differences of included studies between our and previous studies. Supplementary materials 3. Risk assessment of RCTs. Supplementary materials 4. Plot of publication bias. Supplementary materials 5. Plot of sensitivity analysis. Supplementary materials 6. Plot of OS and DFS after omitting some studies. Supplementary materials 7. GRADE analysis of OS and DFS in the HAIC group. [file 12957_2023_3000_MOESM1_ESM.docx]

Supplementary materials 1. Search strategy

| Databases | Search strategy |
| --- | --- |
| Pubmed | #1 **((hepatocellular carcinoma[MeSH Terms]) OR (****hepatocellular carcinoma)) OR (HCC)**  **#2 (****((****hepatic artery infusion chemotherapy) OR (****HAIC)) OR (Transcatheter Arterial Infusion Therapy)) OR (Transarterial Infusion Chemotherapy) OR** **TAI**  **#3 ((((****hepatectomy[MeSH Terms]) OR (****liver resection)) OR (hepatic resection)) OR (****hepatectomy)) OR (****liver surgery)**  **#4** **#1 AND #2 AND #3** |
| **Embase** | **#1** 'hepatic artery infusion chemotherapy'/exp OR (hepatic AND artery AND infusion AND chemotherapy) OR haic OR (transcatheter AND arterial AND infusion AND therapy) OR (transarterial AND infusion AND chemotherapy) OR tai  #2 'liver resection'/exp OR (liver AND resection) OR (hepatic AND resection) OR hepatectomy OR (liver AND surgery)  #3 'liver cell carcinoma'/exp OR (hepatocellular AND carcinoma) OR hcc  **#4 #1 AND #2 AND #3** |
| Web of Science | \| #1 ALL=((hepatocellular  carcinoma)  OR  HCC) \|  \| \| --- \| --- \|   #2 ALL=((hepatic  artery  infusion  chemotherapy)  OR  HAIC  OR  (Transcatheter Arterial Infusion Therapy)  OR  (Transarterial Infusion Chemotherapy)  OR  TAI)  #3 ALL=((liver  resection)  OR  (hepatic resection)  OR  hepatectomy  OR  (liver surgery) )  **#4 #1 AND #2 AND #3** |
| Cochrane library | #1 [Carcinoma, Hepatocellular] explode all trees  #2 (hepatocellular carcinoma) :ti,ab,kw OR (HCC):ti,ab,kw  #3 #1 OR #2  #4 **(hepatic artery infusion chemotherapy)**:ti,ab,kw **OR (HAIC))**:ti,ab,kw **OR (Transcatheter Arterial Infusion Therapy)**:ti,ab,kw **OR (Transarterial Infusion Chemotherapy)**:ti,ab,kw **OR (TAI)**:ti,ab,kw  #5 [Hepatectomy] explode all trees  #6 **(liver resection)**:ti,ab,kw **OR (hepatic resection)**:ti,ab,kw **OR (hepatectomy)** :ti,ab,kw **OR (liver surgery)**:ti,ab,kw  #7 #5 OR #6  #8 #3 AND #4 AND #7 |
| Scopus | #1 ( TITLE-ABS-KEY ( hepatocellular AND carcinoma ) OR TITLE-ABS-KEY ( hcc ) )  #2 ( TITLE-ABS-KEY ( hepatic AND artery AND infusion AND chemotherapy ) OR TITLE-ABS-KEY ( haic ) OR TITLE-ABS-KEY ( transcatheter AND arterial AND infusion AND therapy ) OR TITLE-ABS-KEY ( transarterial AND infusion AND chemotherapy ) AND TITLE-ABS-KEY ( tai ) )  #3 ( TITLE-ABS-KEY ( liver AND resection ) OR TITLE-ABS-KEY ( hepatectomy ) OR TITLE-ABS-KEY ( hepatic AND resection ) OR TITLE-ABS-KEY ( liver AND surgery ) )  **#4 #1 AND #2 AND #3** |
| Ovid | #1 (hepatocellular carcinoma or HCC).af.  #2 ((hepatic artery infusion chemotherapy or HAIC or Transcatheter Arterial Infusion Therapy or Transarterial Infusion Chemotherapy) or TAI).af.  #3 (liver resection or hepatic resection or hepatectomy or liver surgery).af.  **#4 #1 AND #2 AND #3** |

　　 Supplementary materials 2. The differences of included studies between our and previous studies

| Moran *et al* (1) | | |  | Ke *et al* (2) | | |  | Li *et al* (3) | | |  | Present study | | |
| --- | --- | --- | --- | --- | --- | --- | --- | --- | --- | --- | --- | --- | --- | --- |
| Included study | Comparison and Sample size | |  | Included study | Comparison and Sample size | |  | Included study | Comparison and Sample size | |  | Included study | Comparison and Sample size | |
|  | LR+HAIC | LR |  |  | LR+HAIC | LR |  |  | LR+HAIC | LR |  |  | LR+HAIC | LR |
| Niguma 2004 | 6 | 6 |  | Nomami 1991 | 19 | 113 |  | Huang 2015 | 42 | 43 |  | Nomami 1991 | 19 | 113 |
| Kim 2011 | 31 | 62 |  | Niguma 2004 | 6 | 6 |  | Hsiao 2017 | 61 | 160 |  | Kim 2011 | 31 | 62 |
| Nitta 2013 | 38 | 35 |  | Tanaka 2005 | 7 | 8 |  | Nitta 2013 | 38 | 35 |  | Kumatoto 2013 | 16 | 17 |
| Kumamoto 2013 | 16 | 17 |  | Kim 2011 | 31 | 62 |  | Kojima 2015 | 27 | 25 |  | Nagano 2013 | 30 | 20 |
| Nagano 2013 | 30 | 20 |  | Nitta 2013 | 38 | 35 |  | Niguma 2004 | 6 | 6 |  | Nitta 2013 | 38 | 35 |
| Huang 2015 | 42 | 43 |  | Kojima 2015 | 27 | 25 |  |  |  |  |  | Kojima 2015 | 27 | 25 |
|  | LR+I-131 | LR |  | Huang 2015 | 42 | 43 |  |  |  |  |  | Feng 2017 | 42 | 43 |
| Boucher 2003 | 38 | 38 |  | Hsiao 2017 | 61 | 160 |  |  |  |  |  | Hsiao 2017 | 61 | 160 |
| Tabone 2006 | 10 | 20 |  | Hatano 2018 (4) | 134 | 266 |  |  |  |  |  | Hamada 2020 | 37 | 85 |
| Lau 2008 | 21 | 22 |  | Kuramoto 2013 | 6 | 6 |  |  |  |  |  | Hirokawa 2020 | 55 | 59 |
| Chua 2010 | 41 | 41 |  | Li 2020 | 58 | 58 |  |  |  |  |  | Li 2022 | 157 | 158 |
| Chung 2013 | 52 | 51 |  | Hamada 2020 | 37 | 85 |  |  |  |  |  |  |  |  |

1.The study "Hatano 2018" was excluded by us because the data was from 22 institutions between 2001 and 2010 and duplicated with the data from "Kojima 2015". What is more, we do not know whether more data from "Hatano 2018" was duplicated with other studies.

2.The study "Huang 2015" and study "Feng 2017" used the same data and had the same conclusion.

3. The study "Li 2020" is a preliminary report of a RCT and the study "Li 2022" is the final version.

4.In our study, studies with sample size less than 10 in each group were excluded.

Supplementary materials 3. Risk assessment of RCTs

| Study | Random sequence generation | Allocation concealment | Blinding of participants  and personnel | Blinding of outcome assessment | Incomplete outcome data | Selective reporting | Other bias |
| --- | --- | --- | --- | --- | --- | --- | --- |
| Hirokawa 2020 | Low Risk | Low Risk | High Risk | Unclear | Low Risk | Low Risk | Low Risk |
| Li 2022 | Low Risk | Low Risk | High Risk | Unclear | Low Risk | Low Risk | Low Risk |

Supplementary materials 4. Plot of publication bias


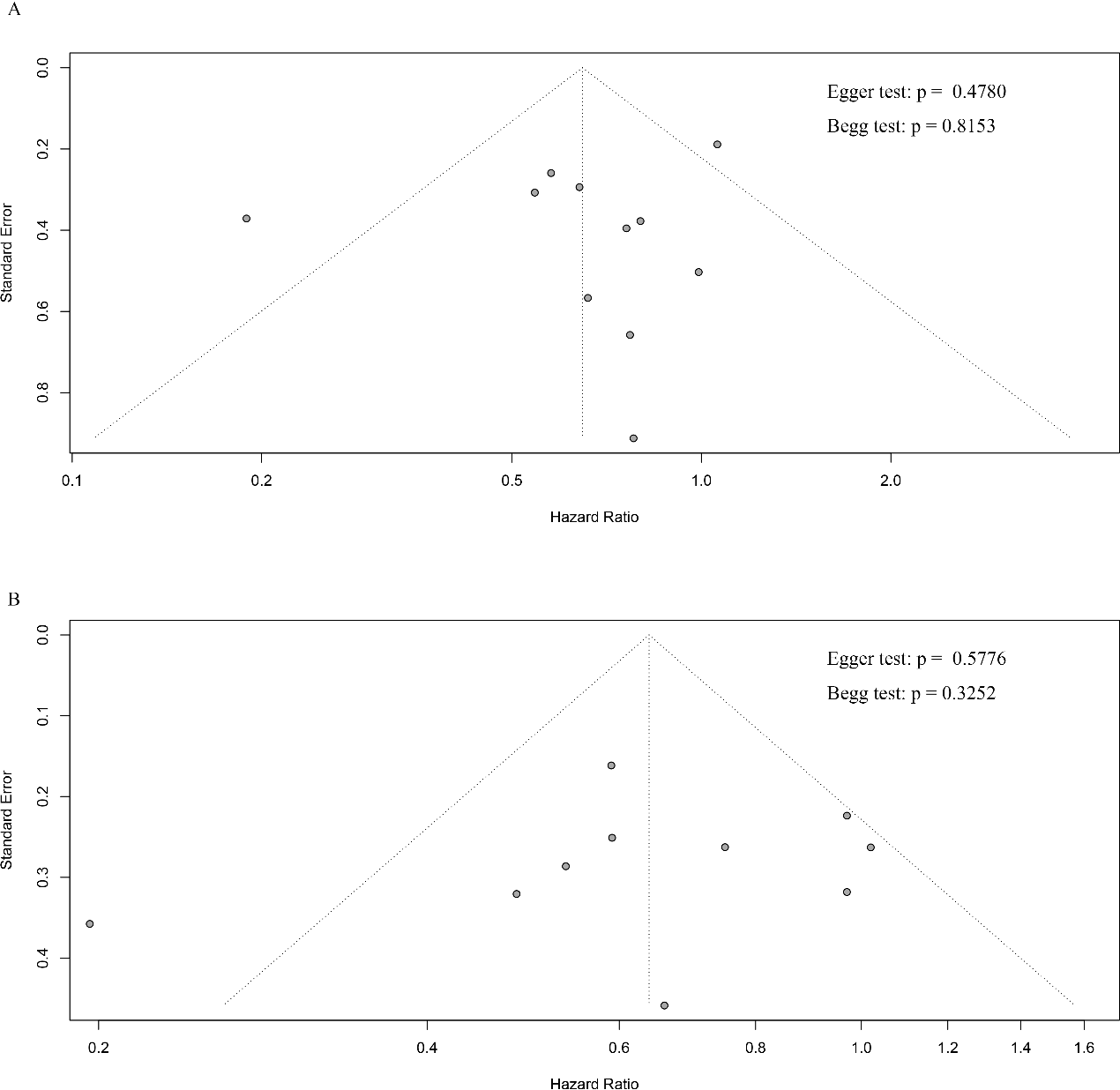


A, is funnel plot of OS; B, is funnel plot of DFS; p values of Egger test and Begg test were at the upper right corner.

Supplementary materials 5. Plot of sensitivity analysis

Sensitivity analysis was conducted by Stata 15 software because R software warninged that "Fisher scoring algorithm did not converge" when conducting sensitivity analysis.


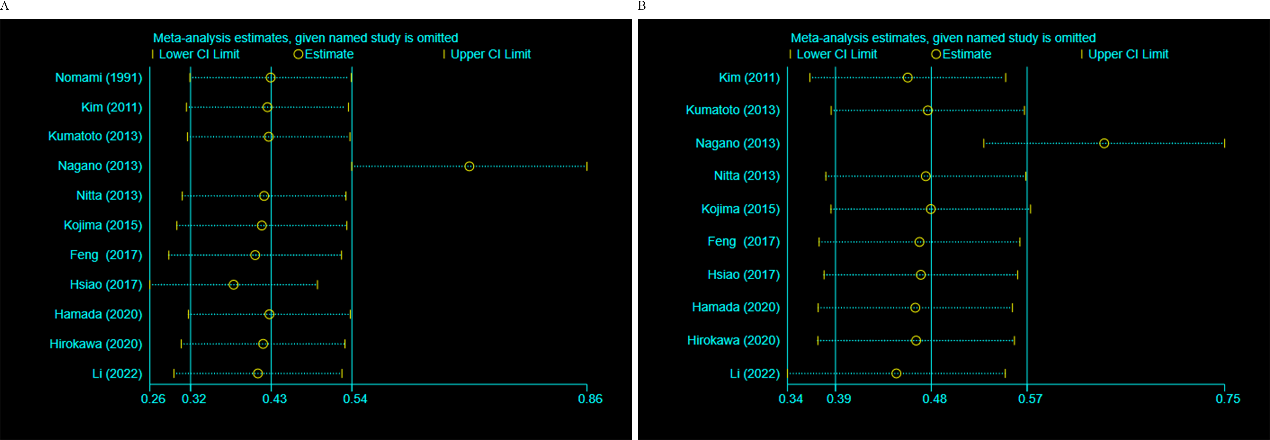


A , plot of sensitivity analysis for OS; B, plot of ensitivity analysis for DFS.

Supplementary materials 6. Plot of OS and DFS after omitting some studies

As a study conducted by Nomami *et al* was published too early in 1991, potential heterogeneity may exist. Sensitivity analysis showed that a certain heterogeneity between the results of "Nagano2013" and other studies. Hence, we re-calculated by omitting study "Nomami 1991" and "Nagano2013" successively and omitting both of them.


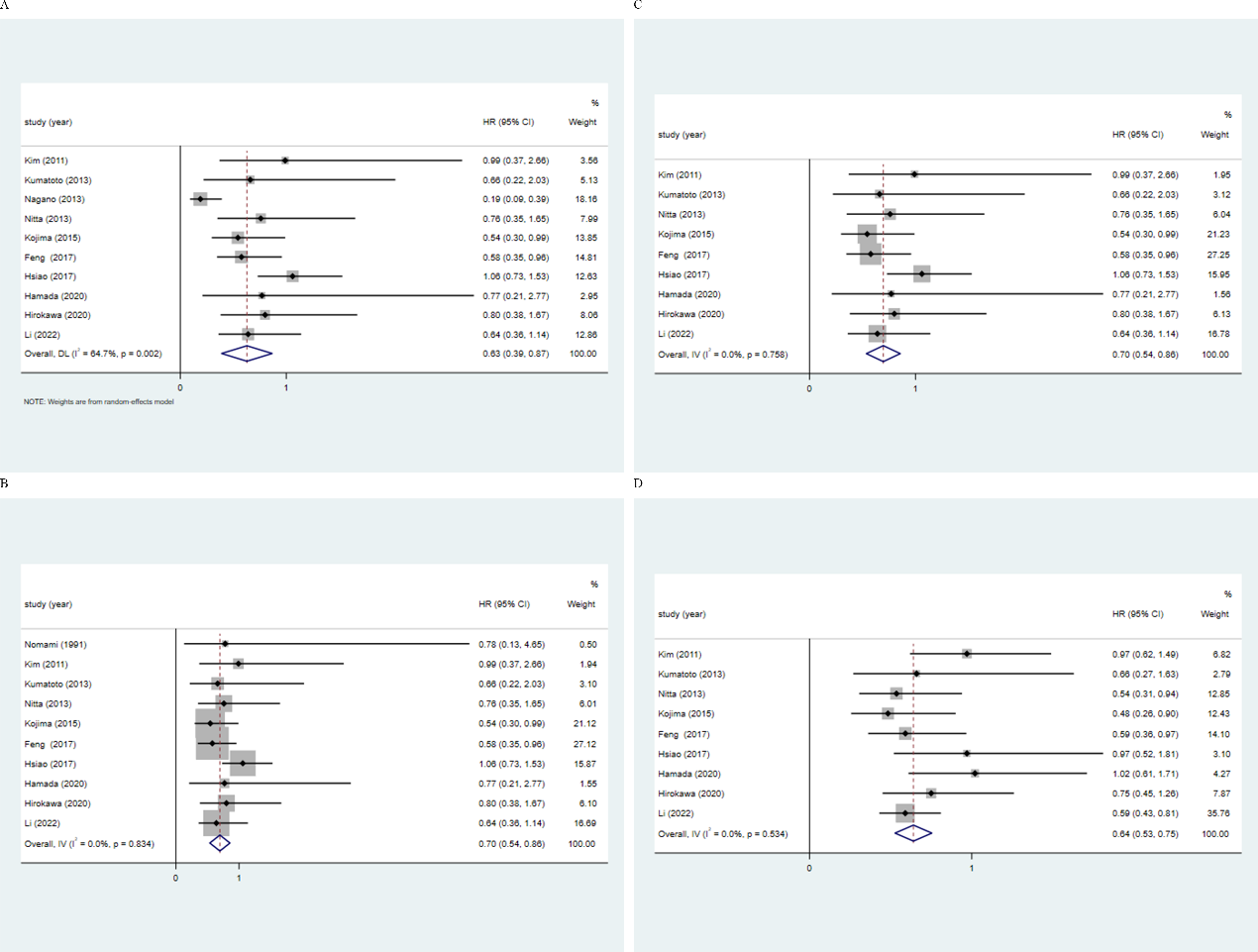
 A, plot of OS after omitting study "Nomami 1991"; B, plot of OS after omitting study "Nagano2013"; C, plot of OS after omitting study "Nomami 1991" and "Nagano2013"; D, plot of DFS after omitting study "Nagano2013".

Supplementary materials 7. GRADE analysis of OS and DFS in the HAIC group

| **HAIC+LR compared to LR for Patients with HCC** | | | | | | |
| --- | --- | --- | --- | --- | --- | --- |
| **Patient or population:** Patients with HCC **Settings:**  **Intervention:** HAIC+LR **Comparison:** LR | | | | | | |
| **Outcomes** | **Illustrative comparative risks* (95% CI)** | | **Relative effect (95% CI)** | **No of Participants (studies)** | **Quality of the evidence (GRADE)** | **Comments** |
|  | Assumed risk | Corresponding risk |  |  |  |  |
|  | **LR** | **HAIC+LR** |  |  |  |  |
| **1-year OS** | **Study population** | | **RR 1.10**  (1.02 to 1.19) | 1290 (11 studies) | ⊕⊝⊝⊝ **very low**^1,2,3^ |  |
|  | **856 per 1000** | **941 per 1000** (873 to 1000) |  |  |  |  |
|  | **Low** | |  |  |  |  |
|  | **300 per 1000** | **330 per 1000** (306 to 357) |  |  |  |  |
|  | **High** | |  |  |  |  |
|  | **975 per 1000** | **1000 per 1000** (994 to 1000) |  |  |  |  |
| **3-year OS** | **Study population** | | **RR 1.29**  (1.10 to 1.52) | 1290 (11 studies) | ⊕⊝⊝⊝ **very low**^1,2,3^ |  |
|  | **616 per 1000** | **795 per 1000** (678 to 937) |  |  |  |  |
|  | **Low** | |  |  |  |  |
|  | **150 per 1000** | **193 per 1000** (165 to 228) |  |  |  |  |
|  | **High** | |  |  |  |  |
|  | **797 per 1000** | **1000 per 1000** (877 to 1000) |  |  |  |  |
| **5-year OS** | **Study population** | | **RR 1.30**  (0.95 to 1.77) | 750 (8 studies) | ⊕⊝⊝⊝ **very low**^1,2,3,4^ |  |
|  | **423 per 1000** | **550 per 1000** (402 to 749) |  |  |  |  |
|  | **Low** | |  |  |  |  |
|  | **50 per 1000** | **65 per 1000** (47 to 88) |  |  |  |  |
|  | **High** | |  |  |  |  |
|  | **678 per 1000** | **881 per 1000** (644 to 1000) |  |  |  |  |
| **1-year DFS** | **Study population** | | **RR 1.24**  (1.08 to 1.43) | 1158 (10 studies) | ⊕⊝⊝⊝ **very low**^1,2,3^ |  |
|  | **593 per 1000** | **736 per 1000** (641 to 849) |  |  |  |  |
|  | **Low** | |  |  |  |  |
|  | **200 per 1000** | **248 per 1000** (216 to 286) |  |  |  |  |
|  | **High** | |  |  |  |  |
|  | **919 per 1000** | **1000 per 1000** (993 to 1000) |  |  |  |  |
| **3-year DFS** | **Study population** | | **RR 1.38**  (1.06 to 1.80) | 1036 (9 studies) | ⊕⊝⊝⊝ **very low**^1,2,3,4^ |  |
|  | **354 per 1000** | **489 per 1000** (375 to 637) |  |  |  |  |
|  | **Low** | |  |  |  |  |
|  | **32 per 1000** | **44 per 1000** (34 to 58) |  |  |  |  |
|  | **High** | |  |  |  |  |
|  | **638 per 1000** | **880 per 1000** (676 to 1000) |  |  |  |  |
| **5-year DFS** | **Study population** | | **RR 1.12**  (0.70 to 1.78) | 595 (6 studies) | ⊕⊝⊝⊝ **very low**^1,2,3,4^ |  |
|  | **333 per 1000** | **373 per 1000** (233 to 593) |  |  |  |  |
|  | **Low** | |  |  |  |  |
|  |  |  |  |  |  |  |
|  | **High** | |  |  |  |  |
|  | **500 per 1000** | **560 per 1000** (350 to 890) |  |  |  |  |
| *The basis for the **assumed risk** (e.g. the median control group risk across studies) is provided in footnotes. The **corresponding risk** (and its 95% confidence interval) is based on the assumed risk in the comparison group and the **relative effect** of the intervention (and its 95% CI).  **CI:** Confidence interval; **RR:** Risk ratio; | | | | | | |
| GRADE Working Group grades of evidence **High quality:** Further research is very unlikely to change our confidence in the estimate of effect.  **Moderate quality:** Further research is likely to have an important impact on our confidence in the estimate of effect and may change the estimate. **Low quality:** Further research is very likely to have an important impact on our confidence in the estimate of effect and is likely to change the estimate. **Very low quality:** We are very uncertain about the estimate. | | | | | | |
| ^1^ Most studies are retrospective studies. ^2^ The results of included studies are inconsistent, the inclusion criteria ara also inconsistent. ^3^ Asymmetry is obvious on funnel plot. ^4^ The 95% confidence interval of RR include no effect and appreciable harm and benefit. | | | | | | |

Refference.

1. Moran A, Ramos LF, Picado O, Pendola F, Sleeman D, Dudeja V, et al. Hepatocellular carcinoma: resection with adjuvant hepatic artery infusion therapy vs resection alone. A systematic review and meta-analysis. J Surg Oncol. 2019;119(4):455-63.

2. Ke Q, Wang L, Wu W, Huang X, Li L, Liu J, et al. Meta-Analysis of Postoperative Adjuvant Hepatic Artery Infusion Chemotherapy Versus Surgical Resection Alone for Hepatocellular Carcinoma. Front Oncol. 2021;11:720079.

3. Li S, Xu J, Zhang H, Hong J, Si Y, Yang T, et al. The Role of Hepatic Arterial Infusion Chemotherapy in the Treatment of Hepatocellular Carcinoma: A Systematic Review and Meta-Analysis. Chemotherapy. 2021:1-10.

4. Hatano E, Uemoto S, Yamaue H, Yamamoto M, Japanese Soc H. Significance of hepatic resection and adjuvant hepatic arterial infusion chemotherapy for hepatocellular carcinoma with portal vein tumor thrombus in the first branch of portal vein and the main portal trunk: a project study for hepatic surgery of the Japanese Society of Hepato-Biliary-Pancreatic Surgery. Journal of Hepato-Biliary-Pancreatic Sciences. 2018;25(9):395-402.
